# Societal and Healthcare Costs of Post-IDFS Events Following Adjuvant Ribociclib Plus an Aromatase Inhibitor (AI) Versus AI Alone in Pre- and Postmenopausal Women with Early HR+/HER2- Breast Cancer in Canada

**DOI:** 10.3390/curroncol33100595

**Published:** 2026-10-01

**Authors:** Dina Soliman, Christine Brezden-Masley, Sandeep Sehdev, Natalia Sabater Anaya, Sophie Boukouvalas, Talitha Vincken

**Affiliations:** 1 Novartis Pharmaceuticals Canada, Montreal, QC H2Y 0C1, Canada; 2 Mount Sinai Hospital, Toronto, ON M5G 1X5, Canada; 3 The Ottawa Hospital Cancer Centre, Ottawa, ON K1H 8L6, Canada; 4 Avalere Health, Washington, DC 20005, USA; 5 Avalere Health, 10672 Athens, Greece; 6 Avalere Health, 3068 AV Rotterdam, The Netherlands

**Keywords:** societal impact, Canada, HR positive, HER2 negative, breast cancer, early breast cancer, recurrence, ribociclib, aromatase inhibitors, aromatase inhibitors

## Abstract

Background: Recent advancements in early breast cancer (EBC) for high-risk populations have significantly improved invasive disease-free survival (IDFS). While cost-effectiveness has been assessed, the broader societal impact of improved IDFS remains unstudied. A non-homogeneous semi-Markov model was developed to estimate total healthcare and societal costs after first-line treatment with ribociclib plus an aromatase inhibitor (AI) or, alternatively, AI alone in patients with hormone-receptor-positive (HR+)/human epidermal growth factor receptor 2-negative (HER2-) EBC. Methods: Using an incidence-based approach, the model calculated total expected annual societal costs of disease-related events after cessation of adjuvant treatment with ribociclib plus AI or AI alone for HR+/HER2- EBC. The model includes five states: IDFS, non-metastatic recurrence (NMR), distant recurrence (DR), secondary primary malignancy (SPM) and death, with a one-year cycle length, and a new cohort entering each year. Health state transition probabilities are based on a previously published cost-effectiveness model (CEM). Costs are tallied only after IDFS (after cessation of adjuvant treatment) and include direct costs of medical care as well as indirect costs related to employment, productivity, and wage loss. Results: At the 4-year NATALEE data cut-off (April 2024), ribociclib plus AI reduced the risk of an IDFS event by 28.5% versus AI alone (HR 0.715), corresponding in the model to 23% fewer non-fatal events (NMR, DR, and SPM), 18% lower mortality, 23% lower total direct costs (−$957,471,465), and 24% lower indirect costs (−$448,542,692) from the period of cessation of adjuvant therapy until death. Healthcare savings were driven primarily by fewer distant recurrences and deaths, while societal savings were largely due to reduced caregiver costs. Conclusions: Treatments that reduce disease recurrence and mortality can significantly lower societal costs and alleviate burden on the healthcare system.

## Simple Summary

Breast cancer recurrence after initial treatment places a significant burden on patients, caregivers, and healthcare systems. While treatments that reduce recurrence risk have been evaluated for their clinical and cost-effectiveness, their broader societal impact—including productivity losses, wage loss, and caregiver costs—has rarely been quantified. This study estimates the total healthcare and societal costs associated with breast cancer recurrence events following adjuvant treatment with ribociclib plus an aromatase inhibitor (AI) compared with AI alone, in pre- and postmenopausal women with hormone-receptor-positive (HR+)/human epidermal growth factor receptor 2-negative (HER2-) early breast cancer (EBC) in Canada. Using an incidence-based semi-Markov model developed in Microsoft Excel^®^, we simulated a cohort of 5851 Canadian patients entering the model annually over a 20-year horizon, tracking transitions across five health states and tallying direct and indirect costs only after completion of adjuvant therapy or disease recurrence. Based on 4-year data from the NATALEE trial, ribociclib plus AI reduced recurrence risk by 28.5% compared with AI alone, which, based on the model estimates, translates into over $1.4 billion in combined direct and indirect lifetime cost savings. These findings highlight the substantial societal value of treatments that reduce disease recurrence in early breast cancer.

## 1. Introduction

In Canada, breast cancer is the most commonly diagnosed cancer among women, with an estimated 31,900 new cases in 2025 alone [1]. On average, 87 women are diagnosed every day, and, each year, approximately 5400 women die from breast cancer, accounting for about 13% of all cancer deaths among women in 2025 [1]. While incidence rates have increased over recent decades—particularly among younger women—mortality rates have declined during the same period [2,3]. These trends highlight both the burden of disease and the progress made in reducing breast cancer mortality through improved awareness, screening, and treatment strategies.

Early breast cancer (EBC) includes Stage 0 and Stages I to III disease. There are three main subtypes of BC, characterized by the presence or absence of hormone receptors—estrogen or progesterone receptors—and by excess or normal levels of human epidermal growth factor receptor 2 (HER2): hormone receptor (HR+)/HER2-, HR-/HER2+, and HR-/HER2-. Within the broader population of breast cancer patients, those with HER2- are more limited in their options for targeted treatment [4]. For patients with HR+/HER2- EBC, treatment options typically include surgery and postoperative radiation therapy. Systemic therapy may be given preoperatively or postoperatively and includes estrogen receptor regulators, aromatase inhibitors (AI), and chemotherapy [5,6].

Recent advancements in EBC that have demonstrated improvements in invasive disease-free survival (IDFS) primarily involve the addition of cyclin-dependent kinase 4/6 (CDK4/6) inhibitors and antibody–drug conjugates (ADCs) for the treatment of high-risk populations (i.e., HR+/HER2-, and HR+/HER2+) [7,8,9,10]. The efficacy and safety of ribociclib plus AI as adjuvant treatment in patients with HR+/HER2- EBC was evaluated in the phase 3 multicenter, randomized, open-label NATALEE trial, which demonstrated a statistically significant improvement in IDFS compared to AI alone [7,11].

Depending on the stage of disease, economic burdens on the patients and their caregivers may vary significantly. The cost of treating breast cancer is significantly lower when the disease is detected early, while expenses rise dramatically as the cancer advances to later stages. Treating a single case of stage IV HR+/HER2- can cost as much as C$ 256,693, mainly due to the high prices of systemic therapy, while managing stage I HR+/HER2- costs only about C$28,201 [12]. These figures highlight the critical importance of early detection and prevention, which can dramatically reduce the financial burden on the healthcare system and improve patient outcomes.

Early treatment is essential not only because of the stark cost differences between stage I and stage IV disease, but also due to the critical need to prevent distant recurrence (DR). Clinically, avoiding DR is vital as patients with a DR face a 5-year survival rate of just 27%, which is considerably lower than survival among patients whose cancer is detected and treated in early stages [13]. Economically, recurrent disease leads to significantly greater healthcare expenditures due to additional drug treatments, hospitalizations, and surgical interventions [14]. Moreover, patients with metastatic breast cancer (mBC) experience substantially worse physical, functional, and emotional well-being than those in remission [15].

The overall societal costs of breast cancer in Canada are significant, arising from patient and caregiver productivity losses, and out-of-pocket payments [16,17]. These costs result from lost work time for both patients and caregivers, household productivity losses, and direct financial expenditures related to care. For example, in Quebec, the average annual wage loss for breast cancer patients has been estimated at over C$12,000 per patient per year, while informal caregiver costs can also be substantial [16]. With few health economics studies available, there is a need to better understand how the prevention of IDFS events—namely recurrence or relapse (RoR)—through add-on therapy to endocrine therapy reduces the societal burden, inclusive of direct and indirect costs. The present study aims to address this gap by estimating downstream healthcare and societal costs associated with breast cancer recurrence events (non-metastatic recurrence [NMR], DR, second primary malignancies [SPM], and death) after completion of adjuvant therapy, among patients with HR+/HER2- EBC among patients who underwent adjuvant treatment with ribociclib plus an aromatase inhibitor (AI) or, alternatively, AI alone, from both the Canadian public payer and societal perspectives.

## 2. Methods

### 2.1. Overview

An economic model was developed to assess the societal impact of recurrence after first-line adjuvant therapy in patients with HR+/HER2- EBC in Canada. Using an incidence-based approach, the model projects the duration of IDFS as well as occurrence and costs of key clinical outcomes after IDFS over a defined time horizon among patients who received adjuvant therapy with ribociclib plus an AI or AI alone. Clinical outcomes after IDFS include NMR, DR, SPM, and death. Costs include direct medical costs and indirect costs such as productivity losses, caregiver expenses, incapacity pension payoffs, and out-of-pocket (OOP) costs.

### 2.2. Model Structure

**Figure 1:**
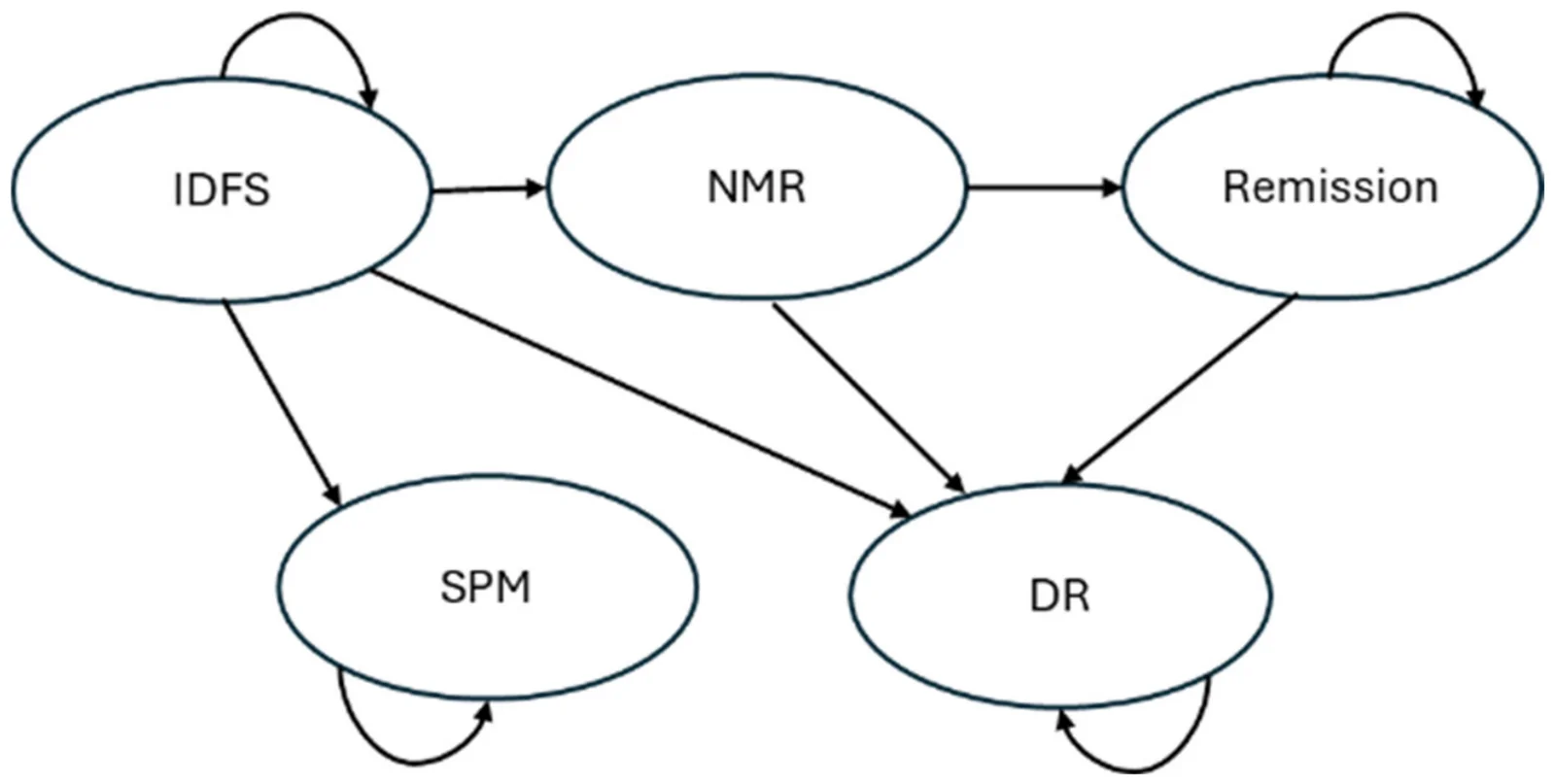
Model structure. Abbreviations: IDFS, invasive disease-free survival; DR, distant recurrence; NMR, non-metastatic recurrence; SPM, secondary primary malignancy.

The societal impact calculator was developed in Microsoft Excel^®^ (Microsoft 365 Apps for enterprise, Version 2608) to evaluate the societal impact of ribociclib plus an AI compared with AI alone. The model uses an incidence-based approach and calculates total expected annual societal impact associated with the risk of breast cancer recurrence after the completion of first-line adjuvant therapy with ribociclib plus an AI in patients with HR+/HER2- EBC, compared with treatment with AI alone. The model is a non-homogeneous, semi-Markov model with five health states: invasive disease-free survival (IDFS), non-metastatic recurrence (NMR), DR, SPM and death (Figure 1). Health state transition probabilities are based on a previously published cost-effectiveness model (CEM), with a new cohort of patients entering the model each year [18]. Event rates are driven by IDFS data from the NATALEE trial [19]. Costs are tallied beginning after transition out of the IDFS state. Accordingly, the model captures the direct and indirect costs associated with NMR, DR, SPM and death; costs of adjuvant treatment (i.e., costs accrued while patients remain in IDFS) are not captured. The model uses a cycle length of one year and a time horizon of 20 years. Costs and health outcomes are discounted at 3.5% per year.

Productivity losses were determined using age- and gender-specific employment rates [20,21]. The model was implemented with the following parameters: 5851 patients entered the model each year, which was calculated as the number of patients with any Stage III and Stage II (N0 with Grade 2–3 or N1+) EBC in the pan-Canadian provinces (see Supplementary Table S1 for details on patient population calculation) [22]. The mean age was 52.8 years, 99.6% were female [18], and patients were followed for 20 years. Patients in the model received adjuvant treatment with ribociclib plus AI or, alternatively, AI alone. Ribociclib plus AI was associated with a 28.5% lower risk of experiencing an IDFS event, based on 4-year data from the NATALEE trial (HR 0.715) [7]. While 5-year NATALEE data are now available, only 4-year data were available at the time of model development and are therefore reported here. All costs were discounted at 3.5% per year.

Table 1 below describes the Population, Intervention, Comparator and Outcome (PICO) of the model along with the model’s perspective and the timeframe.

### 2.3. Population

The population evaluated in the model comprised pre- and postmenopausal women and men with HR-positive, HER2-negative EBC, Stage III, IIB or a subset of Stage IIA cases, after adequate surgical resection, radiotherapy (if indicated), adjuvant or neoadjuvant chemotherapy (if indicated), and who are deemed to be eligible for adjuvant ET for at least 60 months of duration, consistent with the eligibility criteria of the NATALEE trial [9].

### 2.4. Intervention

The intervention evaluated in the model consists of ribociclib, a CDK4/6 inhibitor, in combination with AIs. Ribociclib plus AI was recently approved in Canada as an adjuvant treatment for HR+/HER2- stage II-III EBC. The efficacy and safety of ribociclib plus AI as an adjuvant treatment in patients with HR+/HER2- EBC was evaluated in the phase 3 multicenter, randomized, open-label NATALEE trial [9].

Ribociclib is administered, 400 mg once daily (QD) by mouth (PO) for three weeks followed by one-week break, in a 28-day cycle, for up to 36 months or until disease recurrence or unacceptable toxicity, whichever occurs first.

The AI administered in combination with ribociclib consisted of letrozole 2.5 mg or anastrozole 1 mg, with the addition of goserelin 3.6 mg for men and pre-menopausal women only. AIs were assumed to be received for up to 60 months or until disease recurrence or unacceptable toxicity, whichever occurred first (for detailed regimen dosing assumptions, see Supplementary Table S4).

### 2.5. Comparators

Comparators that comprised AI alone consisted of letrozole (2.5 mg QD PO) or anastrozole (1 mg QD PO), and exemestane (25 mg QD PO) with the addition of goserelin 3.6 mg SC once every 4 weeks (Q4W) for men and pre-menopausal women only. AIs were assumed to be received for up to 60 months or until disease recurrence or unacceptable toxicity, whichever occurred first.

### 2.6. Outcomes

Outcomes evaluated in the model included NMR, DR, SPM and death events for each treatment strategy, as well as the difference in these events between treatment strategies. The model also estimated monetary outcomes, including total direct medical costs, disaggregated direct medical costs, total indirect costs and disaggregated indirect cost, along with the differences in these costs between treatments. Costs were tallied only after transitioning out of the IDFS state. Therefore, the model captures direct and indirect costs associated with NMR, DR, SPM, and death, but costs of adjuvant treatment (i.e., costs accrued while patients remain in IDFS) are not captured.

### 2.7. Model Inputs

Baseline characteristics for the model population were taken from the NATALEE intention-to-treat (ITT) population and are aligned with the previously published CEM inputs [18] (for detailed baseline characteristics, see Supplementary Table S5). Survival curves and transition probabilities were informed by the previously published CEM [18] for ribociclib as adjuvant therapy for HR+/HER2- EBC, while background mortality was sourced from Canadian life tables. Invasive disease-free survival (IDFS) curves used as inputs in the model are presented in Supplementary Figure S4. Key inputs such as patient population estimates, employment rates, wage data, productivity loss, direct medical costs, and indirect costs, including out-of-pocket payments and incapacity payoffs, were obtained from relevant published sources and models. A summary of model inputs is provided in Table 2.

The model accounted for both direct medical costs and indirect societal costs. The estimation of direct medical cost followed the previously published CEM approach and included subsequent therapies, follow-up and monitoring costs, as well terminal care costs [18]. In contrast, societal costs were calculated based on factors such as population employment rates, productivity losses, and average annual wages. Societal costs encompassed patient productivity loss, caregiver productivity loss, incapacity payoffs, and OOP payments. Cost of follow-up services and drug costs are provided in the Supplementary Table S2 and Table S3 respectively.

Societal costs associated with NMR events were calculated by multiplying the number of patients in the NMR state by the proportion of employed individuals in that cohort, the productivity loss in the NMR state relative to the IDFS state, and the annual average wage. A similar approach was used to estimate caregiver productivity loss by applying the caregiver’s productivity decrement instead of the patient’s. Incapacity payoffs were estimated by multiplying the number of NMR patients by the percentage unable to work and the annual incapacity pension amount. OOP payments were determined by multiplying the expected annual OOP costs per NMR patient by the number of patients in the NMR state.

For the calculation of subsequent therapies in the DR health state, the average duration of DR treatment was based on the mean time-to-treatment discontinuation (TTD) of DR, as obtained from the previously published CEM [18]. These costs were applied to the newly incident DR population, calculated as the difference in the number of patients between cycle t + 1 and cycle t. Follow-up and monitoring costs were applied as a one-off cost to the newly incident DR patients. The model allowed for specification of the number of years over which patients accrue follow-up costs, with adjustments made based on the probability of death for DR patients derived from the overall survival (OS) curve. Terminal care costs were also applied as a one-off cost to the newly incident DR patients and adjusted by the proportion of patients who die in the DR state over the specified time horizon. This approach was used because DR was an absorbing state.

### 2.8. Assumptions

The economic models used to inform the population estimates, survival inputs and some of the costs used in this analysis were developed for submission to Canada’s Drug Agency (CDA-AMC), and therefore exclude Quebec in their estimation (Quebec has a distinct health technology assessment agency, the Institut National d’Excellence en Santé et en Services Sociaux (INESSS)). Consequently, the analysis presented is only generalizable to Canadian provinces other than Quebec. The model incorporated state-specific assumptions. In the NMR state, subsequent therapies, which formed the basis of the healthcare cost calculations, were assumed to be taken for one year, and the incapacity pension amount that was used to estimate incapacity payoffs was based on patients being unable to return to work for one year. In the DR state, it was assumed that productivity loss persists for the entire time horizon until retirement. For incapacity payments, patients were assumed not to return to the workforce. For the SPM state, medical follow-up costs were assumed to be incurred for one year following entry into this state. Productivity loss was again assumed to persist until retirement (age 65 years), and incapacity payments were based on patients not returning to work. Productivity losses were applied annually to expected wages and were calculated as a fixed proportion based on the incremental difference between productivity losses at the IDFS state relative to each subsequent health state (e.g., IDFS—30.9%, NMR, DR, SPM—33.3% = 2.4% annual incremental patient productivity loss Table 2). For the purposes of this analysis, the incident and starting populations were assumed to be the same.

## 3. Results

The model demonstrates the potential impact of ribociclib in combination with an AI compared with AI alone on patient outcomes and the associated healthcare and societal costs. Table 3 summarizes the reductions in clinical events and costs estimated by the societal impact calculator.

### 3.1. Results: Clinical Events

Treatment with ribociclib plus AI was associated with a 28.5% reduction in the risk of IDFS events versus AI alone. This was associated with improvements across clinically relevant outcomes, including a 23% reduction in non-fatal events (i.e., NMR, DR and SPM) and an 18% reduction in mortality (Table 3). Non-fatal events avoided by type following a 28.5% decrease in the risk of IDFS are presented in Supplementary Figure S1, while cumulative IDFS events avoided per event type are presented in Supplementary Figure S2.

### 3.2. Results: Healthcare Costs (Payer)

Post-IDFS direct medical costs by health state from the payer perspective for ribociclib plus AI and for AI alone, as well as the incremental values, are presented in Table 3. The reduction in the risk of IDFS events associated with ribociclib plus AI versus AI alone resulted in total post-IDFS payer cost savings of $957,471,465. The majority of these savings were driven by avoided DR events, which accounted for 91% of the payer cost savings ($870,871,089). This reflects the high direct medical cost associated with managing DR relative to other non-fatal events. Avoided NMR and SPM events contributed smaller proportions to the total payer-perspective direct healthcare cost benefits, with savings of $6,119,319 and $118,829, respectively. Cumulative healthcare cost savings are presented in Supplementary Figure S3.

### 3.3. Results: Societal Costs

The calculator also estimated broader post-IDFS societal costs associated with treatment with ribociclib plus AI versus treatment with AI alone (Table 3). The societal costs include medical costs borne by patients (OOP payments), caregiver costs, incapacity pension payoffs and patient productivity losses.

Compared with AI alone, ribociclib plus AI reduced patient productivity losses, caregiver costs, and incapacity pension payoffs by 24%, corresponding to savings of $27,932,263, $307,254,888, and $88,102,796, respectively. OOP payments, representing direct medical costs borne by patients, decreased by 23%, resulting in additional savings of $25,252,745. Overall, these improvements translated into a total societal cost reduction of 24%, resulting in a net saving of $ 448,542,692. In total, these additional costs beyond the payer’s perspective were reduced by $448,542,692. When combined with payer-perspective cost savings, this resulted in total societal cost savings of $1406,014,157 for ribociclib plus AI versus AI alone.

**Figure 2:**
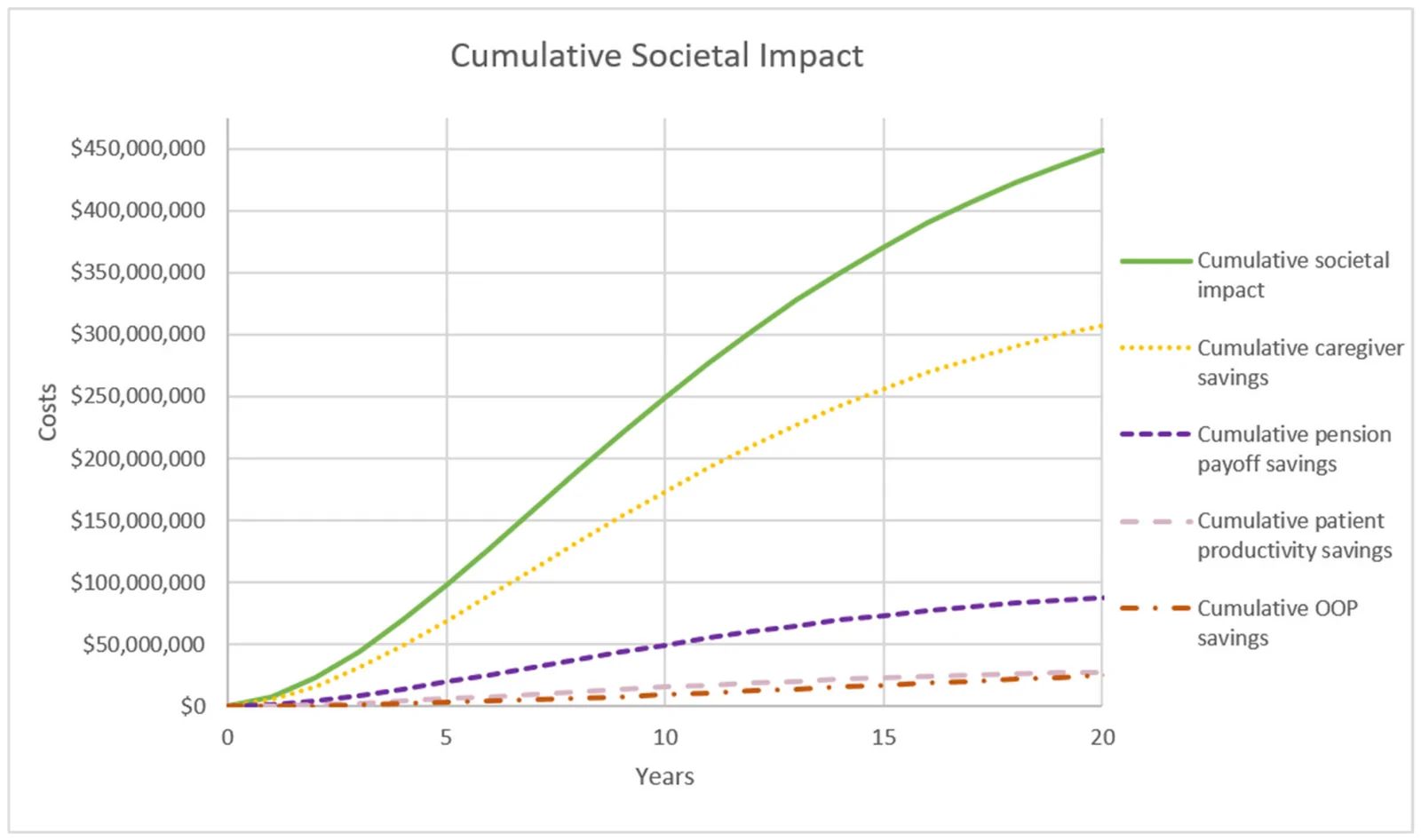
Cumulative cost savings in societal costs. Abbreviations: OOP, out-of-pocket payments.

Figure 2 illustrates the cumulative societal impact, showing how each category of savings contributes to the total societal cost benefit. Notably, cumulative caregiver productivity savings are the largest contributor to overall societal cost savings.

## 4. Discussion

Breast cancer is one of the leading causes of cancer deaths among women in Canada. Although patients diagnosed with HR+/HER2 early breast cancer have some of the best chances of survival relative to breast cancer patients with later diagnoses, they still face a significant risk of disease recurrence with the current standard of care. In this analysis, we demonstrated how the 28.5% reduction in the risk of recurrence estimated in the NATALEE trial of ribociclib in early breast cancer could lead to substantial reductions in disease recurrence, mortality, and both direct and indirect costs, versus AI alone over a 20-year time horizon in Canada. This analysis is focused on the downstream benefits post-adjuvant therapy for EBC and does not account for upfront drug acquisition and administration of adjuvant treatment.

The reduction in invasive disease risk after adjuvant treatment with ribociclib plus AI translated into a 23% reduction in non-fatal events and an 18% reduction in mortality, yielding $957,471,465 in savings from the payer perspective.

Total additional cost savings with the inclusion of societal impact amounted to $1406,014,157. Payer-perspective cost savings were primarily driven by avoided DR events, which accounted for 91% of direct medical cost savings, while caregiver costs represented the single largest component of the additional savings captured in the societal perspective ($307,254,888; approximately 68% of savings beyond the payer perspective).

The reduction in healthcare costs was primarily driven by avoided DR events, which accounted for 91% of payer savings, while caregiver costs represented the single largest component of all savings ($307,254,888; approximately 68% of total societal savings). These findings highlight the importance of early intervention to reduce the risk of recurrence and prevent progression to more costly and burdensome advanced disease states, particularly DR. They also underscore the importance of adopting a societal perspective when evaluating the economic value of interventions that reduce recurrence in early breast cancer, as analyses limited to the payer perspective would substantially underestimate the full economic benefit.

These findings should be considered within the broader landscape of CDK4/6 inhibitors in the adjuvant EBC setting. The monarchE trial demonstrated a significant IDFS and overall survival benefit, with abemaciclib plus endocrine therapy in a high-risk node-positive population [25]. In contrast, palbociclib did not demonstrate IDFS benefit in the PALLAS or PENELOPE-B adjuvant trials [26,27]. Since model development, 5-year follow-up data from the NATALEE trial was reported and have confirmed a sustained reduction in IDFS risk (HR 0.716; 95% CI 0.618–0.829), alongside an emerging overall survival trend (HR 0.800; 95% CI 0.637–1.003) [28]. These updated data support the robustness of the efficacy assumptions underlying the societal cost savings estimated here.

This analysis is subject to certain limitations common to economic models, including potential discrepancies in estimates of effectiveness between clinical trials and real-world evidence, incomplete or inaccurate cost estimates, and variability in patient adherence to treatment. Currently, there are no real-world evidence studies to inform the efficacy of ribociclib plus ET in early breast cancer patients; therefore, estimates from the NATALEE clinical trial represent the most accurate data available for this population. Additionally, these results are not generalizable to all Canadian provinces; Quebec was excluded from the estimation of model inputs as its healthcare system is provincially funded and insured, and the economic models used to inform this analysis were developed for submission to CDA-AMC.

With these limitations in mind, this analysis of the societal impact of reducing the risk of recurrence and death among early breast cancer patients after tumor resection and adjuvant therapy demonstrates meaningful potential to reduce the overall burden of breast cancer on patients and caregivers.

## 5. Conclusions

The societal impact analysis evaluated total healthcare and societal cost savings for patients with HR+/HER2- EBC from the perspective of the Canadian public payer and societal perspectives. The economic model estimated the impact of reducing the risk of recurrence on healthcare and societal costs following the use of ribociclib plus AI compared with AI alone.

Our findings demonstrated that a 28.5% reduction in the risk of IDFS, achieved with ribociclib plus AI compared with AI alone, translates into a 23% reduction in non-fatal events (i.e., NMR, DR, and SPM) and an 18% reduction in mortality. These improvements across key outcomes translated into substantial reductions in total healthcare and societal costs of 23% and 24%, respectively. The reduction in healthcare costs was primarily driven by decreased distant recurrence breast cancer events, while the reduction in societal costs was mainly attributable to decreased caregiver burden.

Overall, advanced treatments such as ribociclib plus AI that improve IDFS have the potential to significantly reduce societal costs and alleviate the burden on the healthcare system.

## Figures and Tables

**Table 1:** PICOT framework.

Component	Approach	
Population	Patients receiving adjuvant treatment for HR+/HER2- high-risk early breast cancer (EBC) who, after surgical resection, had complete tumor removal and met one of the following criteria: Stage IIA with N1, or; Stage IIA with N0 and Grade 3, or; Stage IIA with N0 and Grade 2 with additional high-risk features, defined as at least one of the following: ○ Ki-67 ≥ 20%; ○ Oncotype DX Breast Recurrence Score ≥ 26, or; ○ High risk by genomic risk profiling; ○ Stage IIB, or stage III.	
Intervention	Ribociclib 400 mg plus AI (letrozole 2.5 mg or anastrozole 1 mg) and, for men and pre-menopausal women only, goserelin 3.6 mg.	
Comparators	Aromatase Inhibitors: Anastrozole; Letrozole; Exemestane; Goserelin (for men and pre-menopausal women only).	
Outcomes	Health outcomes: NMR cases avoided; DR cases avoided; SPM cases avoided; Deaths avoided.	Monetary outcomes: Total cost savings; Disaggregated healthcare cost savings; Societal cost savings; Disaggregated societal cost savings.
Costing scope	Costs were applied only after transitioning out of the IDFS state. Therefore, the model captures direct and indirect costs associated with NMR, DR, SPM, and death, but not costs accrued while patients remain in IDFS.	
Perspective	Canadian healthcare system and societal perspective.	
Time horizon	20 years	

Abbreviations: AI, aromatase inhibitor; DR, distant recurrence; DX, diagnosis; EBC, early breast cancer; HER2, human epidermal growth factor receptor; HR, hormone receptor; NMR, non-metastatic recurrence; SPM, secondary primary malignancy; Stage X cancer with N[status]: N[status] indicates the extent of regional lymph node involvement (e.g., N0 = no involvement, N1 = limited involvement, N2+ = involvement of regional lymph nodes classified as N2 or higher).

**Table 2:** Summary of model inputs.

Types of Data	Inputs and Source
Baseline characteristics	NATALEE ITT
Survival curves and transition probabilities Invasive disease-free survival (IDFS); Distributions of IDFS events; NMR transition probability; Distribution of distant recurrence by ET-resistant and ET-sensitive.	Informed by inputs estimated for the ribociclib as adjuvant therapy for HR+/HER2- EBC global cost-effectiveness model (CEM); based on post hoc analyses of NATALEE ITT population data and other published sources for Canada.
Background mortality	Life expectancy and other elements of the complete life table, single-year estimates, Canada, all provinces except Prince Edward Island. Release date: 24 January 2022 Geography: Canada, Province or territory
Incident and cumulative patient population data Annual incident population: 5851; Starting patient population: 5851.	Ribociclib as adjuvant therapy for HR+/HER2- EBC global budget impact model (BIM); estimates of eligible early breast cancer population for Canada excluding Quebec.
Employment rates by age: 15 to 24 years: Women—55.8%, Men—53.9% 25 to 44 years: Women—80.5%, Men—86.7% 45 to 64 years: Women—69.8%, Men—78.3% 65 to 69 years: Women—22.8%, Men—33.4% 70+ years: Women—5.5%, Men—10.9%	Statistics Canada Employment Rates 2024 [21]
Annual wage Women: $51,721.51 Men: $70,054.49	Estimated assuming 50 weeks of work a year using average hourly wages and weekly hours from Statistics Canada [20].
Annual productivity losses and inability to work Patient productivity losses: IDFS—30.9%, NMR, DR, SPM—33.3% Caregiver productivity losses: IDFS—25.9%, NMR, DR, SPM—52.3% Inability to work: NMR—7.0%, DR, SPM—27.0%	Lambert-Obry et al., 2018 [23] Verril et al., 2020 [15]
Direct costs: Drug acquisition; Drug administration; Follow-up and monitoring; Subsequent therapies; Terminal care.	Ribociclib as adjuvant therapy for HR+/HER2- EBC global CEM. Canadian adaptation.
Indirect costs: Monthly OOP payments for non-metastatic breast cancer patients: $225.55 Monthly payoff of incapacity pension: $1210.27	Iragorri et al. [16] Canada Pension Plan: Pensions and benefits monthly amounts [24]

Abbreviations: CEM, cost-effectiveness model; DR, distant recurrence; EBC, early breast cancer; HER2, human epidermal growth factor receptor; HR, hormone receptor; NMR, non-metastatic recurrence; OOP, out-of-pocket; SPM, secondary primary malignancy.

**Table 3:** Results: events and healthcare and societal costs.

Events	Ribociclib + AI	AI	Difference
NMR	3851	5001	−1150
DR	24,069	31,286	−7217
SPM	3667	4762	−1095
Deaths	17,166	20,955	−3789
Direct medical costs borne by payer (healthcare)			
NMR	$20,093,072	$26,212,392	$6,119,319
DR	$2,850,010,053	$3,720,881,142	$870,871,089
SPM	$390,180	$509,009	$118,829
Death	$263,029,018	$343,391,245	$80,362,228
Total	$3,133,522,323	$4,090,993,788	$957,471,465
Indirect societal costs and out-of-pocket payments (societal)			
Patient productivity losses	$87,015,601	$114,947,864	$27,932,263
Caregiver productivity losses	$957,171,614	$1,264,426,502	$307,254,888
Incapacity pension payoffs	$274,464,073	$362,566,869	$88,102,796
OOP payments	$82,697,898	$107,950,643	$25,252,745
Total	$1,401,349,186	$1,849,891,878	$448,542,692
Total costs			
Total healthcare costs	$3,133,522,323	$4,090,993,788	$957,471,465
Total societal costs	$1,401,349,186	$1,849,891,878	$448,542,692
Total costs	$4,534,871,509	$5,940,885,666	$1,406,014,157

Abbreviations: AI, aromatase inhibitor; DR, distant recurrence; NMR, non-metastatic recurrence; OOP, out-of-pocket payments; SPM, secondary primary malignancy.

## Data Availability

Restrictions apply to the availability of these data. Data (NATALEE trial data) were obtained from Novartis and are available from the authors with the permission of Novartis.

## Supplementary Materials

The following supporting information can be downloaded at: https://www.mdpi.com/article/10.3390/curroncol33100595/s1, Figure S1: Non-fatal events avoided by type following a 28.5% decrease in the risk of IDFS; Figure S2: Cumulative IDFS events avoided following a 28.5% decrease in the risk of IDFS; Figure S3: Cumulative cost savings in healthcare costs; Figure S4: Invasive disease-free survival; Table S1: Total eligible population as per the BIA model; Table S2: Costs of follow-up services; Table S3: Drug costs; Table S4: Regimen dosing assumptions; Table S5: Baseline characteristics.
