# Supplementary material for: Societal and Healthcare Costs of Post-IDFS Events Following Adjuvant Ribociclib Plus an Aromatase Inhibitor (AI) Versus AI Alone in Pre- and Postmenopausal Women with Early HR+/HER2- Breast Cancer in Canada
Source: Curr Oncol. 2026 Oct 1;33(10):595. doi: 10.3390/curroncol33100595 (PMC13703256; doi:10.3390/curroncol33100595)
Supplement: Supplementary file 1 [file curroncol-33-00595-s001.zip › curroncol-4461359-supplementary.pdf]

# Supplementary Materials

## Societal and healthcare costs of post-IDFS events following adjuvant ribociclib plus an aromatase inhibitor (AI) versus AI alone in pre and postmenopausal women with early HR+/HER2- breast cancer in Canada

Dina Soliman[1], Christine Brezden-Masley[2], Sandeep Sehdev[3], Natalia Sabater-Anaya[4], Sophia Boukouvalas[5], Talitha Vincken[6]

[1]Novartis Pharmaceuticals Canada, Montreal, Canada; [2]Mount Sinai Hospital, Toronto, Canada; [3]The Ottawa Hospital Cancer Centre, Canada; [4]Avalere Health, Boston, US, [5]Avalere Health, Athens, Greece, [6]Avalere Health, Rotterdam, The Netherlands

S1. Patient population calculation

*Table S1. Total eligible population as per the BIA model*

| | Input Used | Baseline Result | Source |
|---|---|---|---|
| Population Size | | 32,021,468 | Statistics Canada. Population estimates on July 1, by age and gender. Stat Can. (https://www150.statcan.gc.ca/t1/tbl1/en/tv.action?pid=1710000501). Release Date: 2024-02-21 |
| Annual Growth in Breast Cancer Incidence Rate | -0.2% | | Canadian Cancer Statistics Advisory Committee in collaboration with the Canadian Cancer Society, Statistics Canada and the Public Health Agency of Canada. Canadian Cancer Statistics 2021. Toronto, ON: Canadian Cancer Society; 2021. Available at: cancer.ca/Canadian-Cancer-Statistics-2021-EN |
| Annual Breast Cancer Incidence Rate | 73 per 100k | | Canadian Cancer Statistics Advisory Committee in collaboration with the Canadian Cancer Society, Statistics Canada and the Public Health Agency of Canada. Canadian Cancer Statistics 2021. Toronto, ON: Canadian Cancer Society; 2021. Available at: cancer.ca/Canadian-Cancer-Statistics-2021-EN<br><br>Statistics Canada. Population estimates on July 1, by age and gender. Stat Can. (https://www150.statcan.gc.ca/t1/tbl1/en/tv.action?pid=1710000501). Release Date: 2024-02-21 |
| Incident Cases of Breast Cancer | | 23,260 | |
| Incident Cases With HR+/HER2- Tumors | 72.7% | 16,910 | Howlader N, Altekruse SF, Li CI, et al. US incidence of breast cancer subtypes defined by joint hormone receptor and HER2 status. J Natl Cancer Inst. 2014; 106: dju055. DOI: https://doi.org/10.1093/jnci/dju055 |
| Incidence Cases Undergoing Surgery | 86.5% | 14,635 | Sheffield KM, Peachey JR, Method M, et al. A real-world US study of recurrence risks using combined clinicopathological features in HR-positive, HER2-negative early breast cancer. Future Oncol. 2022 Jul;18(21):2667-2682. doi: 10.2217/fon-2022-0310. |
| Any Stage II | 37.0% | 5,415 | Seung SJ, Traore AN, Pourmirza B, Fathers KE, Coombes M, Jerzak KJ. A population-based analysis of breast cancer incidence and survival by subtype in Ontario women. Curr Oncol. 2020;27(2):e191-e198. doi:10.3747/co.27.5769 |
| Stage II With N0 | 51.4% | 2,785 | Fen Ye and Eleanor Sum. NATALEE and MONARCHE eligible population in Real-world: patient characteristics and treatment pattern. Novartis Pharmaceuticals Corp (Data on File); October, 2023. |
| Stage II and N0 with G2/3 Tumors | 52.9% | 1,472 | |
| Stage II With N1 | 48.6% | 2,630 | |
| Stage II and N1 with G3 or ≥5cm Tumor | 25.4% | 668 | |
| Stage II With N2+ | 0.0% | 0 | |
| Any Stage III | 12.0% | 1,750 | Seung SJ, Traore AN, Pourmirza B, Fathers KE, Coombes M, Jerzak KJ. A population-based analysis of breast cancer incidence and survival by subtype in Ontario women. Curr Oncol. 2020;27(2):e191-e198. doi:10.3747/co.27.5769 |

| | | | |
|---|---|---|---|
| Stage III With N1 | 34.9% | 611 | Fen Ye and Eleanor Sum. NATALEE and MONARCHE eligible population in Real-world: patient characteristics and treatment pattern. Novartis Pharmaceuticals Corp (Data on File); October, 2023. |
| Stage III and N1 with G3 or ≥5cm Tumor | 100.0% | 611 | |
| Stage III With N2+ | 61.6% | 1,077 | |
| Any Stage III and Stage II (N0 with Grade 2-3 or N1+) | | 5,851 | |

Abbreviations: HER2-: Human Epidermal Growth Factor Receptor 2 Negative Tumors; HR+: Hormone Receptor Positive; Stage X cancer with N[status]: N[status] indicates the extent of regional lymph node involvement (e.g., N0 = no involvement, N1 = limited involvement, N2+= involvement of regional lymph nodes classified as N2 or higher)

## S2. Model Inputs

*Table S2. Costs of Follow-up Services*

| Service Name | Cost ($CAD) | Cost Estimation |
|---|---|---|
| GP Visit | 79.85 | Based on costs for medical specific assessment by medical oncologists (A443) |
| Mammogram | 68.15 | Based on costs for bilateral mammogram (X185) |
| Oncologist Visit | 79.85 | Based on costs for medical specific assessment by medical oncologists (A443) |
| Echocardiography | 731.80 | Based on costs for Echocardiography (1 and 2 dimensions) (G570 (technical component of $112.60) + G571 (professional component of $96.20) 3.IP.30.DA |
| MUGA scan | 900.60 | Based on costs for nuclear medicine – cardiovascular system – first transit with blood pool images (J867 ($57.30 for procedure + $22.30 for professional fees) 3.IP.70.CC, 3.IP.70.KG) |
| CT scan | 769.95 | Based on costs for CT thorax with IV contrast (X4063.GY.20.WC) |
| Mastectomy | 841.17 | Based on costs for simple mastectomy (# R108) included assistant and anesthesiologist unit fees and time units with the assumption of a 3 hour surgery duration. |
| Breast Reconstruction | 1189.61 | Based on costs for breast skin reconstruction (#R118) with breast mound creation by prosthesis (#E529) including assistant and anesthesiologist unit fees and time units, assuming a 4 hr procedure duration |
| Multidisciplinary care | 166.5 | Based on costs for oncologist consultation (A445) |
| Radiotherapy | 1087.50 | Mittman et al. 2014 [31] |
| Complete blood count | 3.98 | Based on costs for complete blood count (L393) |
| Electrocardiogram | 415.15 | Assumed same as ribociclib metastatic evaluation, adjusted to 2023 CAD |
| Oncologist consultation | 166.50 | Assumed same as multidisciplinary care |
| Serum chemistry | 3.98 | Assumed same a complete blood count |
| X-ray | 471.50 | Assumed same as ribociclib metastatic evaluation, adjusted to 2023 CAD |
| Liver function test | 17.43 | Based on costs of liver function tests for codes used in ribociclib metastatic evaluation: L700, L222, L223, L191, L005, L029 |

Source: Ontario Schedule of Benefits 2023

Abbreviations: CAD: Canada; CT: Computed Tomography; GP Visit: General Practitioner Visit; MUGA scan: Multi-gated Acquisition Scan

*Table S3. Drug Costs*

| Treatment | mg per unit | Units per pack | Cost per pack |
|---|---|---|---|
| Ribociclib | 200 | 42 | $94.13 |
| Letrozole | 2.5 | 30 | $1.38 |
| Anastrozole | 1 | 30 | $0.95 |
| Abemaciclib | 150 | 28 | $111.86 |
| Exemestane | 25 | 30 | $1.33 |
| Tamoxifen | 20 | 30 | $0.35 |
| Fulvestrant | 500 | 2 | $58.29 |
| Palbociclib | 100 | 21 | $253.91 |
| Everolimus | 10 | 30 | $172.26 |
| Capecitabine | 150 | 60 | $0.46 |

Source: IQVIA Health Care Database

*Table S4. Regimen dosing assumptions*

| Treatment | Component | Health State | Administration type | Dosing units | Dose | Administrations per cycle | Length of cycle (days ) | Percent receiving |
|---|---|---|---|---|---|---|---|---|
| Letrozole [32] | | NMR | Oral | mg | 2.5 | 28 | 28 | 69% |
| Anastrozole [32] | | NMR | Oral | mg | 1 | 28 | 28 | 32% |
| Ribociclib + Fulvestrant [33] | Ribociclib | DR | Oral | mg | 600 | 21 | 28 | 4% |
| | Fulvestrant | DR | Injection | mg | 500 | 1 | 28 | |
| Palbociclib + Fulvestrant [34] | Palbociclib | DR | Oral | mg | 125 | 21 | 28 | 3% |
| | Fulvestrant | DR | Injection | mg | 500 | 1 | 28 | |
| Abemaciclib + Fulvestrant | Abemaciclib | DR | Oral | mg | 300 | 28 | 28 | 4% |
| | Fulvestrant | DR | Injection | mg | 500 | 1 | 28 | |
| Ribociclib + NSAI [32] | Ribociclib | DR | Oral | mg | 600 | 21 | 28 | 12% |
| | Letrozole | DR | Oral | mg | 2.5 | 28 | 28 | |
| Palbocilib + NSAI [35] | Palbociclib | DR | Oral | mg | 125 | 21 | 28 | 28% |
| | Letrozole | DR | Oral | mg | 2.5 | 28 | 28 | |
| Abemaciclib + NSAI [36] | Abemaciclib | DR | Oral | mg | 300 | 28 | 28 | 12% |
| | Anastrozole | DR | Oral | mg | 1 | 28 | 28 | |
| | Letrozole | DR | Oral | mg | 2.5 | 28 | 28 | |
| Letrozole [32] | | DR | Oral | mg | 2.5 | 28 | 28 | 6% |

| | | | | | | | | |
|---|---|---|---|---|---|---|---|---|
| Anastrozole [32] | | DR | Oral | mg | 1 | 28 | 28 | 2% |
| Everolimus + Exemestane | Everolimus | DR | Oral | mg | 10 | 28 | 28 | 4% |
| | Exemestane | DR | Oral | mg | 25 | 28 | 28 | |
| Fulvestrant [33] | | DR | Injection | mg | 500 | 1 | 28 | 6% |
| Exemestane [37] | | DR | Oral | mg | 25 | 28 | 28 | 3% |
| Tamoxifen [38] | | DR | Oral | mg | 20 | 28 | 28 | 5% |
| Capecitabine [39] | | DR | Oral | mg/m2 | 2500 | 14 | 21 | 12% |

*Table S5: Baseline characteristics*

| **Parameter** | **Value** | **Source** |
|---|---|---|
| Median age (years) | 52.80 | NATALEE trial [18] |
| % female | 99.60 | |
| Mean weight (kg) | 72.30 | |
| Mean BSA | 1.78 | |

Abbreviations: BSA, body surface area

Model outcomes

*Figure S1: Non-fatal events avoided by type following a 28.5% decrease in the risk of IDFS*

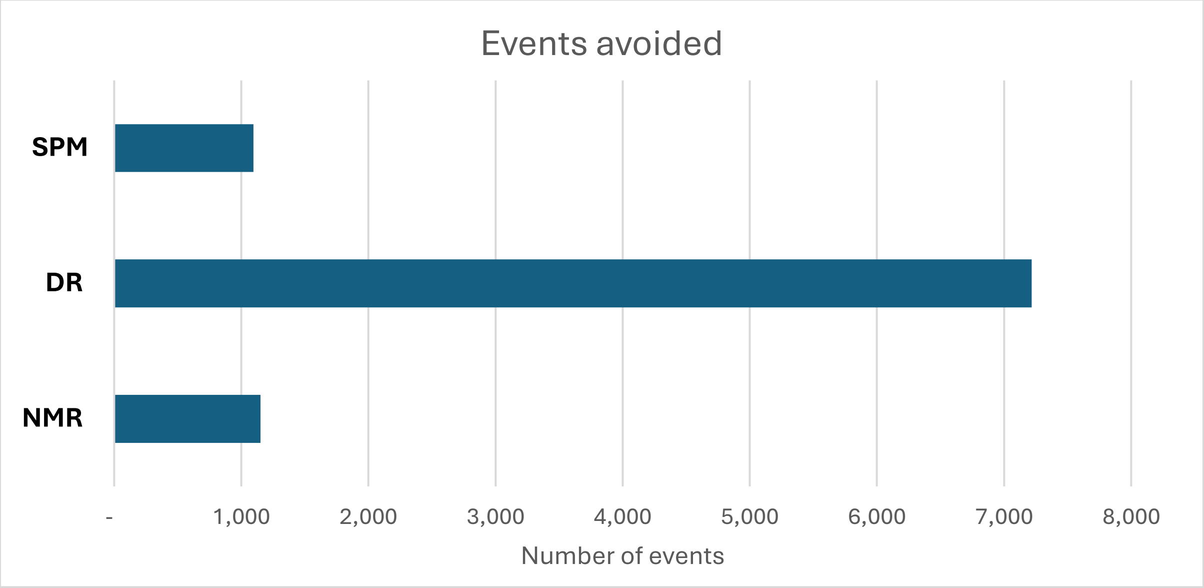


Abbreviations: DR, distant recurrence; NMR, non-metastatic recurrence; SPM, secondary primary malignancy

*Figure S2: Cumulative IDFS events avoided following a 28.5% decrease in the risk of IDFS*

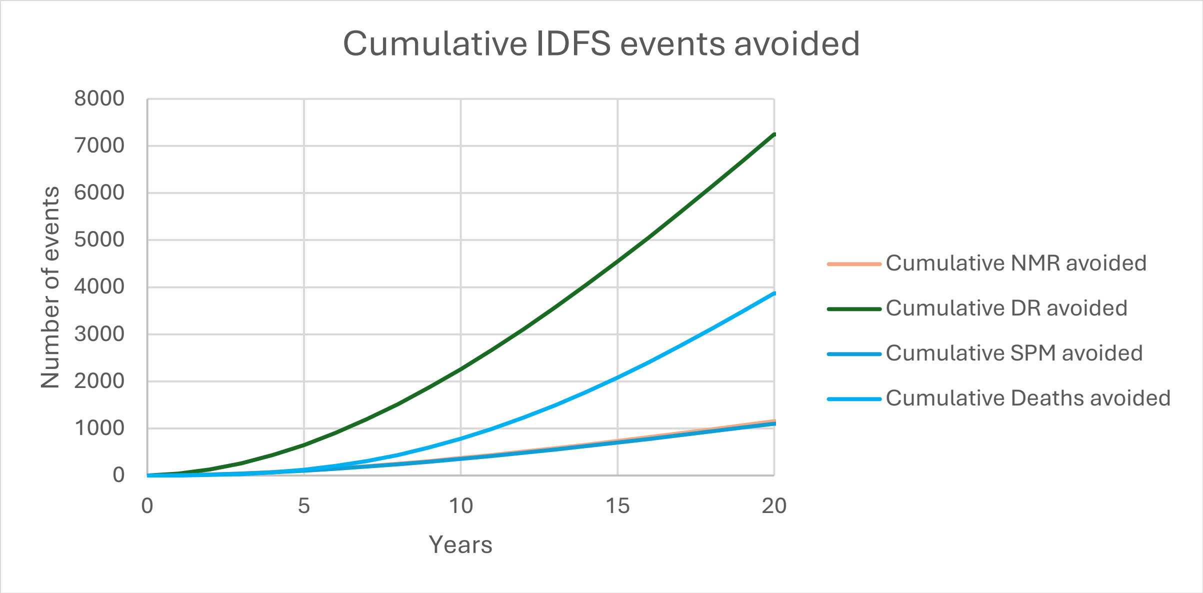


Abbreviations: DR, distant recurrence; IDFS, invasive disease-free survival; NMR, non-metastatic recurrence; SPM, secondary primary malignancy

*Figure S3: Cumulative cost savings in healthcare costs*

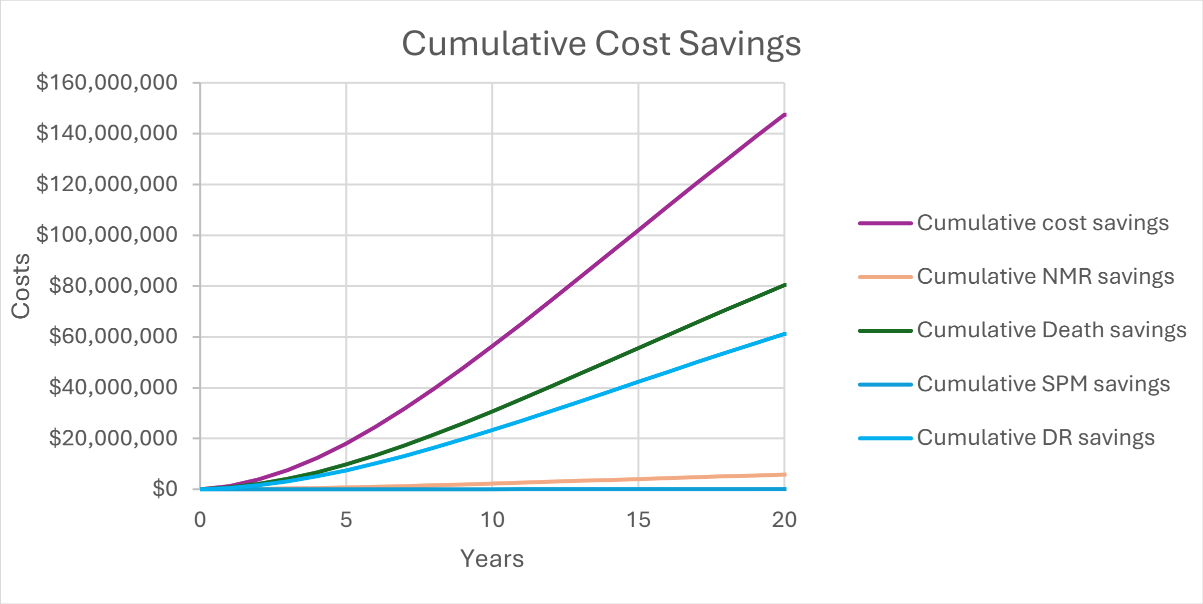


Abbreviations: DR, distant recurrence; NMR, non-metastatic recurrence; SPM, secondary primary malignancy

*Figure S4: Invasive Disease-Free Survival*

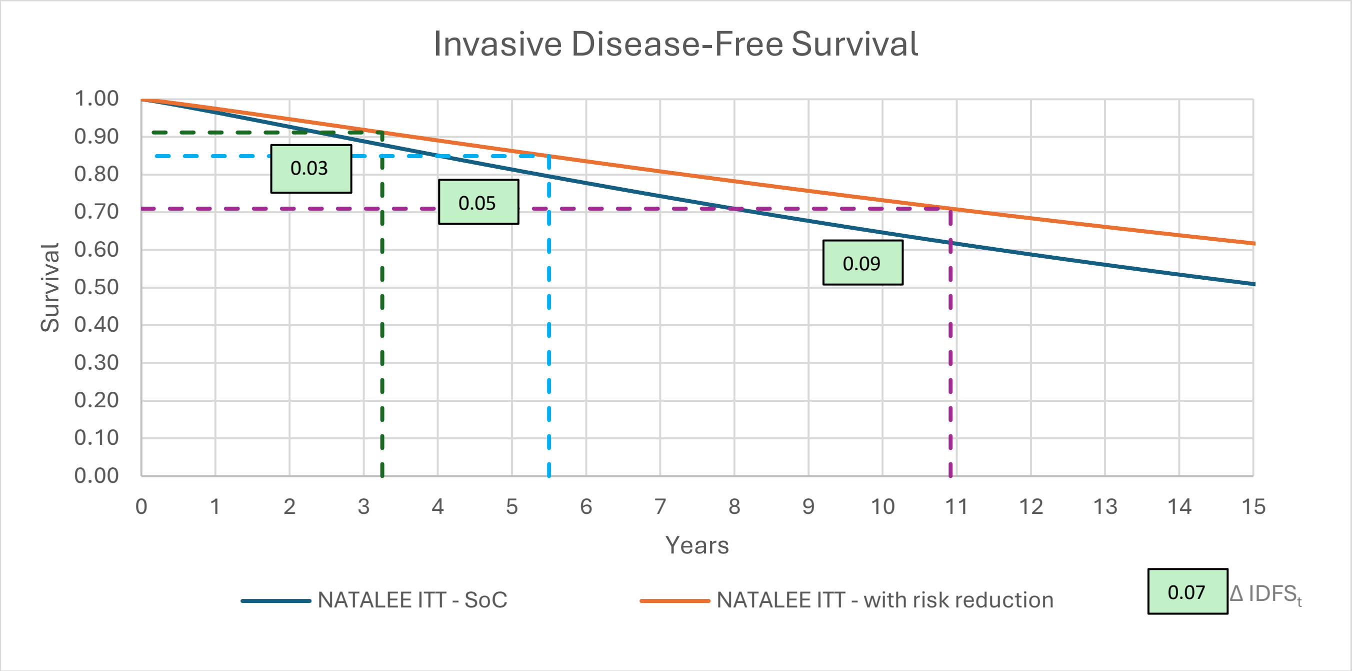


Abbreviations: IDFS, invasive disease-free survival; NMR, non-metastatic recurrence; SPM, secondary primary malignancy
